# CD147 Promotes CXCL1 Expression and Modulates Liver Fibrogenesis

**DOI:** 10.3390/ijms19041145

**Published:** 2018-04-10

**Authors:** Wen-Pu Shi, Di Ju, Hao Li, Lin Yuan, Jian Cui, Dan Luo, Zhi-Nan Chen, Huijie Bian

**Affiliations:** 1 Department of Cell Biology, National Translational Science Center for Molecular Medicine, State Key Laboratory of Cancer Biology, Fourth Military Medical University, Xi’an 710032, China; shiwenpu126@163.com (W.-P.S.); lhcerc@fmmu.edu.cn (H.L.); cuij93@163.com (J.C.); luodan997@163.com (D.L.); zhinanchen@fmmu.edu.cn (Z.-N.C.); 2 Department of Physiology, Basic Medical College, Shaanxi University of Chinese Medicine, Xianyang 712046, China; judi5211314@163.com; 3 Clinical Laboratory, No. 457 Hospital of PLA, Wuhan 430000, China; yuanlin1987gnu@163.com

**Keywords:** CD147, CXCL1, hepatic stellate cells, fibrosis

## Abstract

Activated hepatic stellate cells (HSCs) release pro-inflammatory and pro-fibrogenic factors. CXC chemokine-ligand-1 (CXCL1) is expressed on HSCs. We previously found that the CD147 is overexpressed in activated HSCs. In this study, we showed an important role of CD147 in promoting liver fibrosis by activating HSCs and upregulating expression of chemokines. Specifically, we found that CD147 specific deletion in HSCs mice alleviated CCl_4_-induced liver fibrosis and inhibited HSCs activation. Overexpression of CD147 upregulated the secretion of CXCL1. Meanwhile, CXCL1 promoted HSCs activation through autocrine. Treating with PI3K/AKT inhibitor could effectively suppress CD147-induced CXCL1 expression. Taken together, these findings suggest that CD147 regulates CXCL1 release in HSCs by PI3K/AKT signaling. Inhibition of CD147 attenuates CCl_4_-induced liver fibrosis and inflammation. Therefore, administration of targeting CD147 could be a promising therapeutic strategy in liver fibrosis.

## 1. Introduction

Liver fibrosis can be caused by hepatitis B virus or hepatitis C virus infection, alcohol, non-alcoholic fatty liver disease/non-alcoholic steatohepatitis, and other relatively rare conditions, such as autoimmune hepatitis, Wilson’s disease, and primary/secondary biliary cholangitis. Sustained chronic liver injury leads to normal cellular functional disruption and imbalance of the degradation and synthesis of extracellular matrix (ECM), which eventually develops the liver fibrosis [1]. Hepatic stellate cells (HSCs) exist in the space between parenchymal cells and liver sinusoidal endothelial cells of the hepatic lobule. Following chronic liver injury, HSCs activate into α-SMA^+^ contractile myofibroblast-like cells, which are characterized by the increased proliferation and migration, the unbalanced matrix degradation and the release of pro-inflammatory and pro-fibrogenic factors [2].

HSCs also have immunological functions—they express functional chemokine receptors and chemokines, including CXCL1, CXCL8, CXCL9, CXCL10, CCL2, CCL3 and CCL5 [3]. CXC chemokine-ligand-1 (CXCL1), also known as Gro-alpha, is a ligand for G-protein-coupled receptor CXCR2. CXCL1 is considered to be involved in the activation of HSCs [4], fibrogenesis and angiogenesis [5,6]. CXCL1 has also been recognized as a biomarker associated with hepatocellular carcinoma in a serum level [7].

CD147 is a transmembrane glycoprotein and a member of immunoglobulin superfamily. CD147 is widely expressed on numerous cells in carcinoma leading to the invasion, proliferation and survival of cancer cells, which is an important marker of tumor and poor prognosis [8,9,10]. Our previous studies showed that transforming growth factor-β1-CD147 positive feedback loop promoted the activation of HSCs, and the expression of CD147 in liver fibrosis and cirrhosis was increased which was positively correlated with the Child–Pugh grade [11,12].

In this study, we show that CD147 activates HSCs and upregulates CXCL1 expression in HSCs. In line with this, HSCs-specific CD147-knockout mice have greatly reduced HSCs activation and CXCL1 expression, leading to attenuated liver fibrosis. CD147 regulates CXCL1 expression in HSCs via PI3K/AKT pathway.

## 2. Results

### 2.1. CXCL1 Expression Was Increased in Activated HSCs

**Figure 1:**
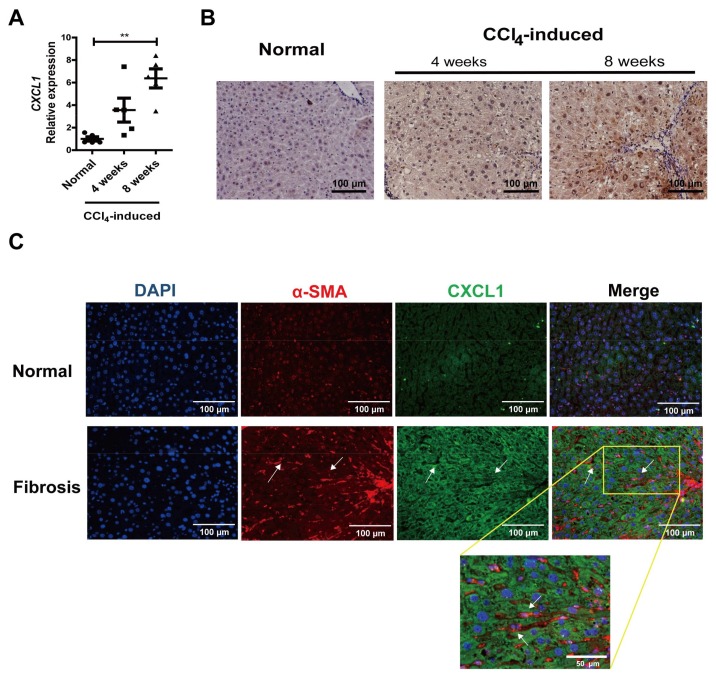
CXCL1 expression increased in liver fibrosis. (**A**) Real-time RT-PCR detection of hepatic gene expression of *CXCL1* in normal control and carbon tetrachloride (CCl_4_)-treated mice (*n* = 5). *Glyceraldehyde-3-phosphate dehydrogenase (GAPDH)* was used as the normalization control, ** *p* < 0.01; (**B**) Immunohistochemistry analysis of CXCL1 in liver tissues from CCl_4_-induced mice; (**C**) Immunofluorescence detection of CXCL1 and α-SMA in liver tissues from normal control and CCl_4_-induced mice (eight weeks). Arrows indicate CXCL1 (green) expression in the activated hepatic stellate cells (HSCs), which are positive for α-SMA (red).

It was reported that CXCL1 expression is upregulated in CCl_4_-induced liver injury [5]. We examined CXCL1 level in liver tissues from normal controls and CCl_4_-induced mice. The *CXCL1* expression showed a time-dependent increase in liver fibrotic tissues (Figure 1A). CXCL1 can be produced by liver cells, like hepatocytes and HSCs. Immunohistochemistry and immunofluorescence revealed that CXCL1 expressed in both hepatocytes and non-parenchymal liver cells (Figure 1B,C). Furthermore, an increase of CXCL1 cytoplasm expression was observed in the activated HSCs that were positive for α-SMA in CCl_4_-induced mouse liver tissues (Figure 1C).

### 2.2. CXCL1 Promoted HSCs Activation and Co-Localized with CD147 in HSCs

**Figure 2:**
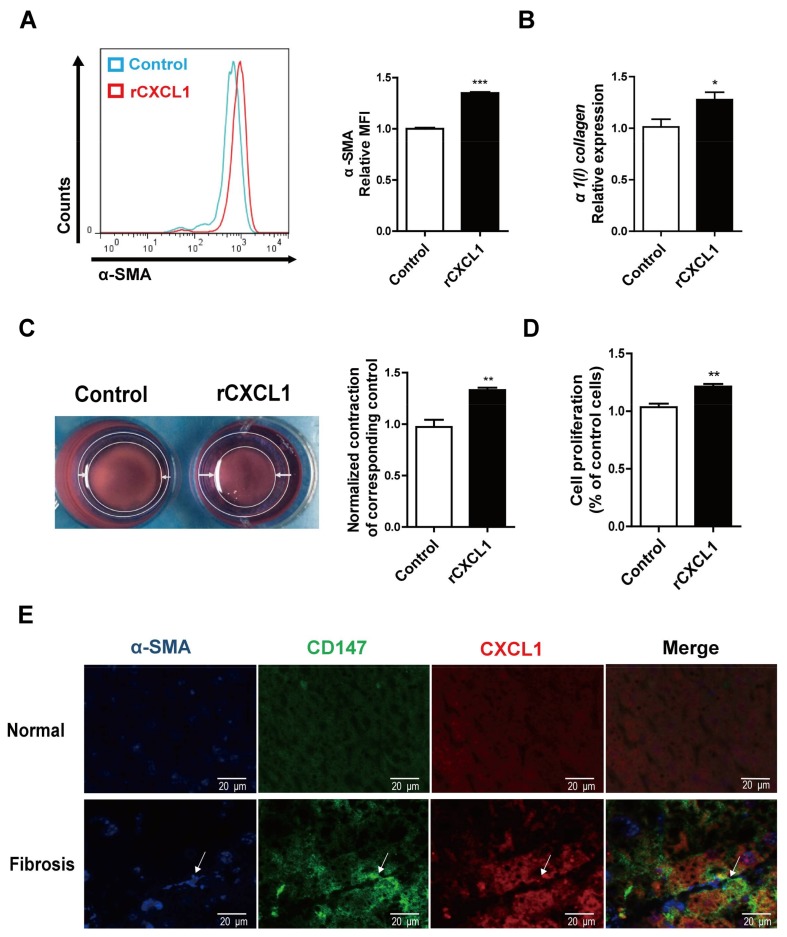
CXCL1 promoted HSCs activation and co-localized with CD147 in HSCs. (**A**) Flow cytometry analyses of α-SMA expression; (**B**) Real-time RT-PCR detection of *α1(I) collagen* mRNA level. *GAPDH* was used as the normalization control; (**C**) Representative phase contrast images and quantitative analysis of collagen-based cell contraction; (**D**) CCK-8 assay; (**E**) Immunofluorescence detection of CD147, CXCL1 and α-SMA in liver tissues from normal control and CCl_4_-induced mice (eight weeks, *n* = 5). Arrows indicate α-SMA (blue), CD147 (green), and CXCL1 (red) expression in the activated HSCs. LX-2 cells were treated with 100 ng/mL rCXCL1 for 24 h. The results were shown as the mean ± SD. * *p* < 0.05, ** *p* < 0.01, *** *p* < 0.001.

To evaluate the effect of CXCL1 on HSCs activation, LX-2 cells were treated with human recombinant CXCL1 (rCXCL1) for 24 h and subjected to detection of α-SMA and type I collagen expression. Fluorescence activated cell sorting (FACS) and RT-PCR analysis showed that the expressions of α-SMA and *α1(I) collagen* were increased with rCXCL1 stimulation (Figure 2A,B). Cell contraction assay demonstrated that the surface area of gel was decreased (Figure 2C), indicating the cell intensive contraction after rCXCL1 treatment. The proliferation of LX-2 cells was also promoted as measured with CCK-8 assay (Figure 2D). Meanwhile, the activated HSCs showed both higher CD147 and CXCL1 expression (Figure 2E). Taken together, these results indicate that rCXCL1 promotes the activation phenotypes of HSCs.

### 2.3. Generation of HSCs-Specific CD147-Knockout Mice

**Figure 3:**
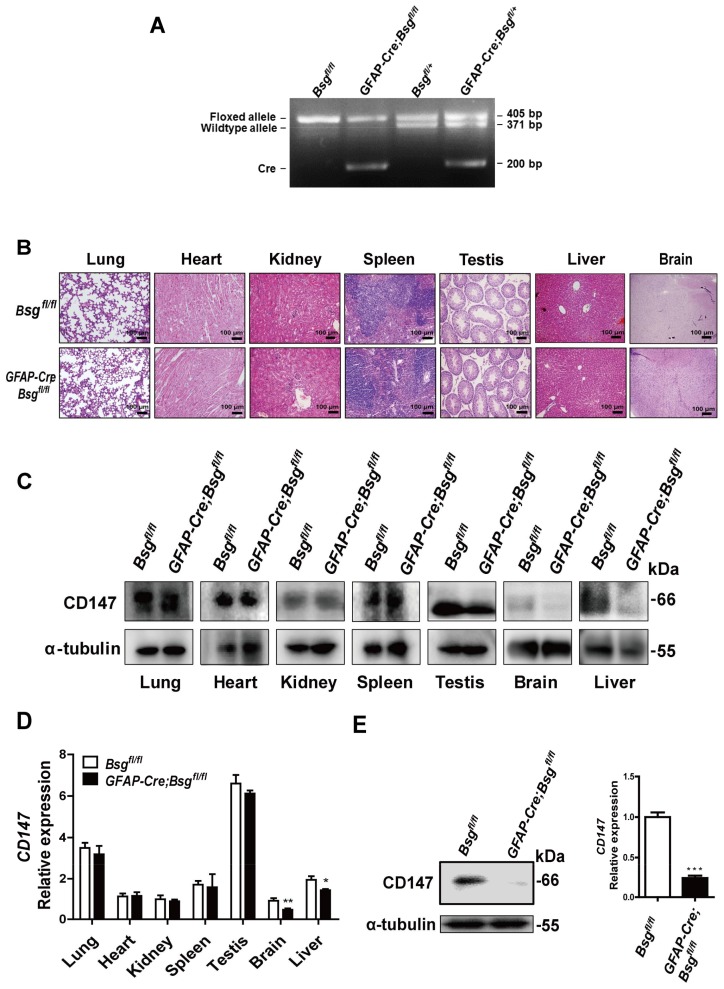
Generation of HSCs-specific CD147-knockout mice. (**A**) Identification of specific knockout of *CD147* gene in the mouse genome; (**B**) HE stain of different tissues in *Bsg^fl/fl^* and *GFAP-Cre;Bsg^fl/fl^*; (**C**) Western blot; and (**D**) real-time RT-PCR analysis of CD147 in different tissues; (**E**) Western blot and real-time RT-PCR analysis of CD147 expression in mouse primary HSCs. *GAPDH* was used as the normalization control. The results were shown as the mean ± SD. *n* = 3. * *p* < 0.05, ** *p* < 0.01, *** *p* < 0.001.

We hypothesize that CD147 regulates the CXCL1 expression in HSCs. To obtain HSCs-specific CD147-knockout mice, we crossed the conditional CD147 targeting mice (*Bsg^fl/fl^*) [13] with the *GFAP-Cre* transgenic mice. Four types of transgenic mice *Bsg^fl/+^*, *Bsg^fl/fl^*, *GFAP-Cre;Bsg^fl/fl^* and *GFAP-Cre;Bsg^fl/+^* were generated (Figure 3A). The *GFAP-Cre;Bsg^fl/fl^* and *Bsg^fl/fl^* mice were used for the following experiments. Histological analysis revealed that *GFAP-Cre;Bsg^fl/fl^* mice showed no spontaneous lesions in lung, heart, kidney, spleen, testis, liver and brain (Figure 3B). It was reported that GFAP mainly expresses on astrocytes in the central nervous system, while also expressing in the cartilage cells, fibroblast, hepatic epithelial cells and HSCs [14,15,16]. The *GFAP-Cre;Bsg^fl/fl^* mice showed the lower expression of CD147 in brain and liver both in the mRNA and protein levels, while there was no such significant change in other tissues (Figure 3C,D). The primary HSCs were then isolated to further verify the specific knockout of CD147 in mouse HSCs. Western blot and RT-PCR analysis showed that the expression of CD147 in isolated HSCs from *GFAP-Cre;Bsg^fl/fl^* mice was significantly reduced compared with that of *Bsg^fl/fl^* mice (Figure 3E).

### 2.4. CD147 Deletion in HSCs Alleviated CCl_4_-Induced Liver Fibrosis and Deregulated CXCL1 Expression

**Figure 4:**
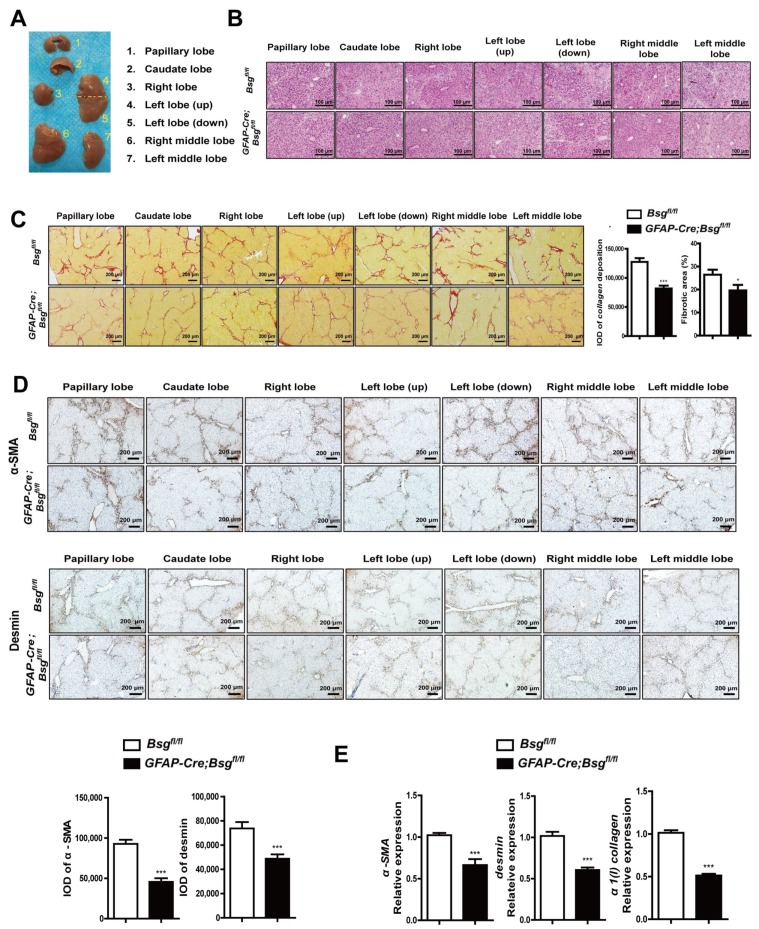
HSCs-specific deletion of CD147 alleviated CCl_4_-induced liver fibrosis *Bsg^fl/fl^* and *GFAP-Cre;Bsg^fl/fl^* mice treated with CCl_4_ for eight weeks (*n* = 4). (**A**) Representative photograph of seven parts of the mouse liver according to the anatomical structure; (**B**) Representative microphotograph of HE-stained paraffin-embedded sections of liver tissues; (**C**) Representative microphotograph of sirius red-stained paraffin-embedded sections of liver tissues (left), statistical analysis of integrated option density (IOD) and expression area percentage of sirius red collagen deposition (right); (**D**) Representative microphotograph of immunohistochemistry analysis of α-SMA and desmin (up), statistical analysis of IOD of α-SMA and desmin (down); (**E**) Real-time RT-PCR detection of hepatic gene expression of *α-SMA*, *desmin* and *α1(I) collagen*. *GAPDH* was used as the normalization control. The results were shown as the mean ± SD. * *p* < 0.05, *** *p* < 0.001.

The *GFAP-Cre;Bsg^fl/fl^* and *Bsg^fl/fl^* mice were subjected to CCl_4_ intraperitoneal injection for induction of liver fibrosis. According to the anatomical structure, the mouse liver was divided into the papillary lobe, caudate lobe, right lobe, left lobe (up), left lobe (down), right middle lobe, and left middle lobe (Figure 4A). The histological images showed that *Bsg^fl/fl^* mice had obvious pseudolobule and infiltration of inflammatory cells, whereas *GFAP-Cre;Bsg^fl/fl^* mice showed attenuated pseudolobule coupled with the reduced infiltration of inflammatory cells and liver damage (Figure 4B). The collagen was stained with sirius red, and the expression intensity and the percentage of positive expression area were statistically analyzed. As shown in Figure 4C, *GFAP-Cre;Bsg^fl/fl^* mice demonstrated the decreased collagen synthesis compared with that of *Bsg^fl/fl^* mice. These results suggest that the knockout of CD147 in HSCs inhibits collagen deposition and liver injury, alleviating the development of fibrosis.

We used the immunohistochemistry to detect the activated HSCs marker, as shown in Figure 4D, histological assessment showed a significant reduction of α-SMA and desmin expression in *GFAP-Cre;Bsg^fl/fl^* mice. Meanwhile, the hepatic *α-SMA*, *desmin*, and *α1(I) collagen* gene expression were also inhibited (Figure 4E). These results suggest that HSCs’ CD147 specific knockout inhibits HSCs activation during CCl_4_-induced fibrosis.

**Figure 5:**
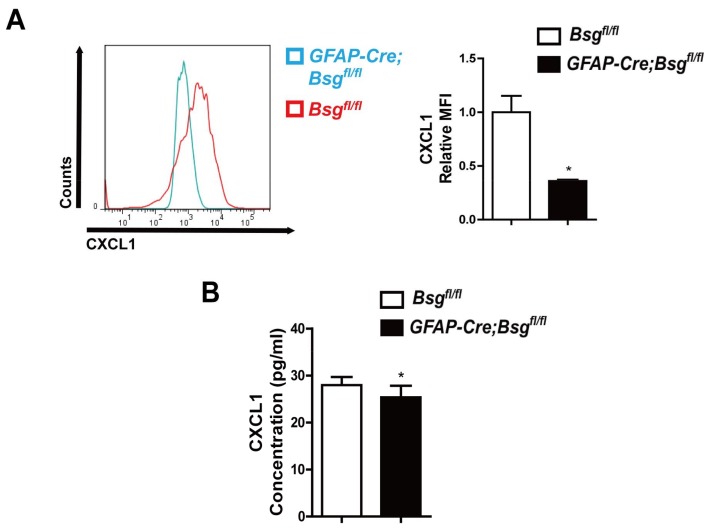
HSCs-specific deletion of CD147 deregulated CXCL1 expression. (**A**) Fluorescence activated cell sorting (FACS) analysis of CXCL1 expression in primary HSCs isolated from *Bsg^fl/fl^* and *GFAP-Cre;Bsg^fl/fl^* mice (*n* = 3); (**B**) Enzyme-linked immunosorbent assay (ELISA) detection of serum CXCL1 in CCl_4_-induced *Bsg^fl/fl^* and *GFAP-Cre;Bsg^fl/fl^* mice (eight weeks, *n* = 4). The results were shown as the mean ± SD. * *p* < 0.05.

Then we examined CXCL1 level in primary mice HSCs without CCl_4_ induced by FACS. The relative median fluorescence intensity (MFI) of CXCL1 in primary *GFAP-Cre;Bsg^fl/fl^* mouse HSCs was reduced (Figure 5A). In addition, serum CXCL1 level was decreased in CCl_4_-induced *GFAP-Cre;Bsg^fl/fl^* mice as detected by enzyme-linked immunosorbent assay(ELISA) (Figure 5B).

### 2.5. CD147-Regulated CXCL1 Expression in HSCs via the PI3K/AKT Pathway

**Figure 6:**
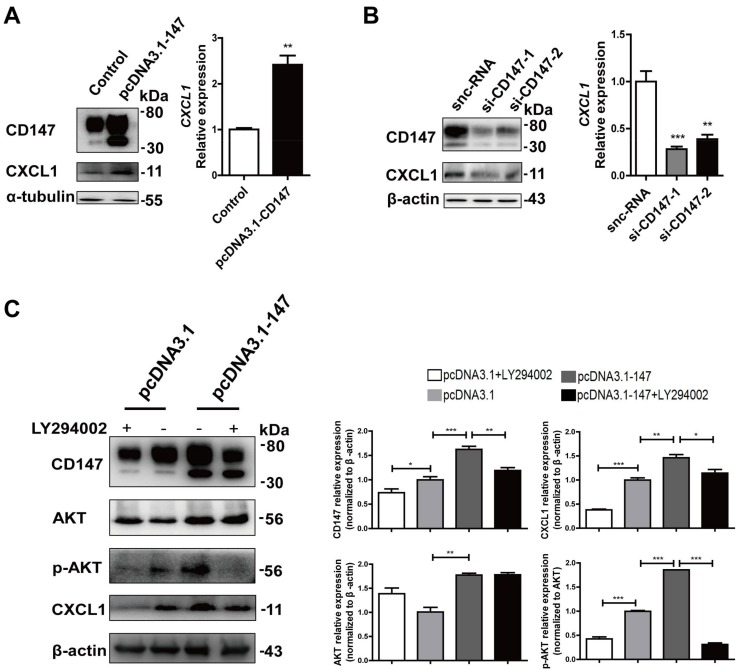
CD147 regulated CXCL1 expression in HSCs via PI3K/AKT pathway. (**A**) Western blot and real-time RT-PCR analysis of CD147 and CXCL1 in LX-2 cells that transfected with pcDNA3.1-CD147; (**B**) Western blot and real-time RT-PCR analysis of CD147 in LX-2 cells transfected with CD147 siRNA. LX-2 cells were transfected with sncRNA as the control; (**C**) Western blot analysis of CD147, AKT, p-AKT, and CXCL1 in pcDNA3.1-CD147-transfected LX-2 cells that were pre-incubated with 10 μmol/L LY294002. Western densitometry was performed for three independent experiments. Cells were transfected with pcDNA3.1(+) as the control. *GAPDH* was used as the normalization control. The results were shown as the mean ± SD. * *p* < 0.05, ** *p* < 0.01, *** *p* < 0.001.

Transient over-expression of CD147 increased the CXCL1 expression in LX-2 cells (Figure 6A). Next, we evaluated the effects of silencing CD147 in LX-2 cells by siRNA. Knockdown of CD147 by si-CD147 decreased the expression of CXCL1 compared with that of silencer-negative control siRNA (snc-RNA) (Figure 6B). These results demonstrate that CD147 promotes CXCL1 expression in HSCs, which is consistent with the previous hypothesis. To investigate whether CD147 regulated CXCL1 through PI3K/AKT pathway, we treated LX-2 cells with 10 μmol/L LY294002, a PI3K inhibitor. The results showed that CD147 overexpression induced the AKT phosphorylation (p-AKT) and elevated CXCL1 expression. Meanwhile, the CD147-induced CXCL1 expression was significantly inhibited by the selective FAK/PI3K inhibitor LY294002 (Figure 6C). All of these results indicated the crucial role of activated PI3K/AKT signaling in CD147-regulated CXCL1 expression.

## 3. Discussion

Patients with fibrosis often result from different etiologies of chronic liver injury. Cirrhosis is the terminal stage of progressive liver fibrosis, eventually developing to the hepatocellular carcinoma. Liver fibrosis is shown to be a reversible process [17,18,19], withdrawal of the chronic injury results in decrease of pro-inflammatory and fibrogenic cytokines, increased collagenase activity, decreased ECM production, and disappearance of hepatic-activated HSCs [20,21,22]. Our previous results show that CD147 upregulates fibrosis-related marker α-SMA and *α1(I) collagen* in HSCs, and the positive loop of transforming growth factor-β1-CD147 promoted the activation of HSCs [11,12]. Here, we provided additional evidence that knockout CD147 in mouse HSCs inhibited collagen deposition, inflammatory infiltration and HSCs activation, alleviating CCl_4_-induced liver fibrosis. In addition, we found that overexpression of CD147 in LX-2 cells upregulated expression of chemokine CXCL1, which was upregulated in activated HSCs. Moreover, CXCL1 exerted a positive activation loop on HSCs, enhancing HSCs activation phenotypes. Taken together, these findings support our conclusions that CD147 stimulates the release of chemokine CXCL1 and promotes the development and progression of liver fibrosis.

Accumulated evidence suggests that chemokines play a critical role in acute and chronic liver diseases, mediating the immune infiltration in injured liver. Additionally, chemokines can also directly affect the biology of liver resident cells, such as HSCs, during inflammatory and fibrogenic tissue responses [23]. It is reported the increased hepatic expression of pro-inflammatory genes *TNF*, *EMR1*, *CCL2*, *MPO*, *CXCL1* and *CXCL2* in liver injury [24]. In our study, we found that CXCL1 in activated HSCs was upregulated in CCl_4_-induced fibrosis. CXCL1 binds to the CXCR2, which expresses on HSCs and neutrophils, and several types of cancer including melanoma [25], lung [26] and pancreatic cancers [27]. Stimulation of HSCs with recombinant CXCL1 results in increased collagen type I and α-SMA production [5,28]. Similarly, in our study rCXCL1 also upregulated the activation markers, α-SMA and *α1(I) collagen* in LX-2 cells and promoted the cell contraction and proliferation.

We used the transgenic mouse *GFAP-Cre;Bsg^fl/fl^* to further verify the CD147 function in HSCs in our study. Liver injury is usually accompanied with inflammatory response. A high-fat diet combined with acute ethanol consumption synergistically induces acute liver inflammation and injury via upregulation of hepatic CXCL1 expression and promotion of hepatic neutrophil infiltration [29]. In CCl_4_-induced mouse fibrosis, *GFAP-Cre;Bsg^fl/fl^* mice showed less hepatic inflammatory infiltration and serum CXCL1 expression. We had proven that overexpression of CD147 promoted *α1(I) collagen* synthesis, while *GFAP-Cre;Bsg^fl/fl^* mice showed less amount of collagen deposition. Liver fibrosis is due to the unbalanced degradation and synthesis of ECM. Activated HSCs are a major source of collagen and ECM and play a key role in the formation of hepatic fibrosis [30,31]; quantitative assessment of HSCs activity is one of the means to detect the development of fibrosis. The *GFAP-Cre;Bsg^fl/fl^* mice showed a reduction of α-SMA and desmin expression, which indicates that inhibiting CD147 contributes to the HSCs deactivation and, thus, the reversal of HSC activation contributes to termination of fibrogenesis [32].

The activated HSCs have activation signatures (MAPK and PI3K-AKT phosphorylation) and upregulated genes (*COL1A1*, α-*SMA*, and *CXCL1*) expression [33]. As integrin is important for HSCs transdifferentiation [34], in integrin-mediated signaling transduction process, FAK is a key molecule and acts like an integrator that accepts and amplifies signals in the cell and activates downstream signal pathway [35]. CD147 can bind to integrin and activates the downstream FAK/PI3K signaling pathway [36]. CD147 overexpression induced the AKT phosphorylation; however, treating with FAK/PI3K inhibitor LY294002, CD147-induced AKT phosphorylation and CXCL1 expression were significantly inhibited. Taken together, CD147 mediated CXCL1 expression probably by directly binding to integrin in HSCs through the PI3K/AKT cell signal activation.

Liver fibrosis shows complex interplay between the epithelial cells, inflammatory cells, myofibroblasts and ECM components of the wound-healing response [2]. In summary, we demonstrate that CD147 promotes the CXCL1 expression and modulates the liver fibrosis. Therapeutically, our results strongly suggest a potential for inhibition of CD147 as a treatment strategy in liver fibrosis.

## 4. Materials and Methods

### 4.1. Cells Culture and Reagents

Human HSCs cell line, LX-2 was cultured in Dulbecco’s Modified Eagle Medium (DMEM) (Hycolon, Logan, UT, USA) supplemented with antibiotics (100 U/mL of penicillin and 100 mg/mL of streptomycin) and 10% fetal bovine serum in a humidified atmosphere containing 5% CO_2_ at 37 °C. Mouse primary HSCs were isolated from the mice livers mainly by in situ pronase/collagenase perfusion of mouse liver, followed by density gradient-based separation, as described [37]. The cells were cultured in DMEM (Hycolon) supplemented with antibiotics (100 U/mL of penicillin and 100 mg/mL of streptomycin) and 15% fetal bovine serum. Human recombinant CXCL1 was from Peprotech (Princeton, NJ, USA). PI3K/AKT inhibitor, LY294002 was from Cell Signaling Technology (Danvers, MA, USA).

### 4.2. Mice

All experimental protocols were approved by the Laboratory Animal Ethics Committee of the Fourth Military Medical University (No. 20121003, 16 October 2012) and performed in strict accordance with the People’s Republic of China Legislation Regarding the Use and Care of Laboratory Animals. HSCs-specific Cre recombinase transgenic mice (glial fibrillary acidic protein (*GFAP*)*-Cre*) purchased from Shanghai Biomodel Organism Science & Technology Development Co., Ltd. (Shanghai, China) and conditional CD147 targeting mice (*Bsg^fl/fl^*) were constructed by Dr. Yao Hui [13] in our laboratory. All gene identification primers were synthesized by the Beijing Genomics Institute (BGI, Beijing, China) and listed in Table 1. To induce chronic liver injury, mice were treated with 10% carbon tetrachloride (CCl_4_) intraperitoneally at a dose of 5 μL/g, three times per week.

### 4.3. siRNA Transfection

Silencing was performed by the transient transfection of siRNA oligos using Lipofectamine 2000 (Invitrogen, Carlsbad, CA, USA) following the manufacturer’s instructions. We used two silencing oligos from GenePharma (Shanghai, China): si-*CD147*-1: 5′-GTA CAA GAT CAC TGA CTC T-3′; si-*CD147*-2: 5′-GTT CTT CGT GAG TTC CTC-3′.

### 4.4. Western Blot

Western blot was performed as previously described [11]. The primary antibodies used were mouse anti-human CD147 antibody (1:1000) prepared by our laboratory [38], anti-AKT (1:2000; ARE6004), anti-phospho-AKT (1:500; ARE6002) were purchased from Antibody revelation (San Diego, CA, USA), rat anti-mouse CD147 (1:500; ab34016) were purchased from Abcam (Cambridge, UK), anti-β-actin (1:1000; M1210-2) were purchased from Hubio (Hangzhou, China), and anti-α-tubulin (1:1000; 66031-1-Ig) were purchased from Proteintech (Wuhan, China). A Western-Light chemiluminescent detection system (Image Station 4000 MM Pro, XLS180, Kodak, Rochester, NY, USA) was used to visualize the signals.

### 4.5. Real-Time PCR

First, total RNA was extracted by a Total RNA Kit II (Omega, Riverside, CA, USA), then using a PrimeScript™ RT reagent kit (TaKaRaBio, Otsu, Japan) reverse transcript to cDNA. All the steps were based on the product protocol. Single-stranded cDNA was amplified by quantitative RT-PCR using a SYBR Premix ExTaq™ kit (TaKaRaBio) on a Stratagene Mx3005P™ Real-Time PCR System (Agilent Technologies, Waldbronn, Germany). Glyceraldehyde-3-phosphate dehydrogenase (GAPDH) mRNA was used to normalize RNA inputs. RT reactions were performed as previously described [11]. All primers were synthesized by the Beijing Genomics Institute (BGI, Beijing, China) and listed in Table 1.

### 4.6. Enzyme-Linked Immunosorbent Assay (ELISA)

First, we used serum separator tube and allowed mice blood samples to clot for one hour at room temperature, then centrifuged for 15 min at approximately 3000× *g*, at 4 °C. Serum CXCL1 detection was performed using a CXCL1 enzyme-linked immunosorbent assay kit (Mlbio, Shanghai, China) according to the manufacturer’s instructions. The concentration in each sample well was determined by interpolation from a standard curve. Each sample was tested in triplicate.

### 4.7. Cell Contraction Assay

We used a standard kit assay (Cell Biolabs, San Diego, CA, USA), operated according to the manufacturer’s instructions. The gel area was quantified by ImageJ software (ImageJ 1.48, National Institutes of Health). The gel contraction area was calculated by the initial gel area (outer white circle) minus the terminal gel area (inner white circle). The normalized contraction of corresponding control was a ratio of control contraction area to rCXCL1 contraction area.

### 4.8. Collagen Staining

Collagen accumulation was detected with sirius red, which was performed as previously described [37]. Liver tissues were fixed with 4% formalin and embedded in paraffin. Following deparaffinization and hydration, the sections were stained with sirius red (Sigma, Darmstadt, Germany).

### 4.9. Immunohistochemistry

Liver tissues were fixed with 4% formalin and embedded in paraffin. Sections were deparaffinized and incubated with primary antibodies including anti-CXCL1 (1:100; ab86436), anti-α-SMA (1:100; ab7817), and anti-desmin (1:100; ab86592) (Abcam, Cambridge, UK). Staining was performed as described previously [39].

### 4.10. Immunofluorescence

Immunofluorescence was carried out as described previously [39]. Primary antibodies included anti-α-SMA (1:100; ab7817), anti-CXCL1 (1:100; ab86436) and anti-CD147 antibodies (1:100; ab34016) (Abcam, Cambridge, UK).

### 4.11. Fluorescence Activated Cell Sorting (FACS)

FACS analysis was carried out as described previously [40]. Primary antibodies included anti-α-SMA (1:100; ab7817) (Abcam, Cambridge, UK) and anti-CXCL1 (1:100; MAB4542) (RD, Minneapolis, MN, USA). The cells were analyzed by flow cytometry (BD FACSCalibur™ Flow Cytometer, BD Bioscience, San Jose, CA, USA) using FlowJo software (FlowJo 7.6, BD Bioscience).

### 4.12. CCK-8

LX-2 cells (5 × 10^4^ cells/well) were seeded in 96-well plates and incubated with 100 ng/mL rCXCL1 for 24 h. Ten µL of the CCK8 (Engreen, Beijing, China) was added to each well using the pipettor. The cells were incubated for 2 h. The absorbance of each sample was measured using a microplate reader at a wavelength of 450 nm.

### 4.13. Statistical Analysis

Each experiment was repeated at least three times. Student’s *t*-test was used to compare the two mean values. A one-way analysis of variance was performed to compare the multiple mean values. Data were presented as the mean ± SD from three independent experiments unless otherwise indicated. The Graphpad Prism software and SPSS 17.0 software were used for statistical analysis. A *p* value < 0.05 was considered statistically significant.

## 5. Conclusions

In summary, we demonstrate that CD147 promotes the CXCL1 expression in HSCs and modulates liver fibrosis. Therapeutically, our results strongly suggest a potential for inhibition of CD147 as a treatment strategy in liver fibrosis.

## Figures and Tables

**Table 1:** Sequences of PCR primers.

Gene	Forward Sequence (5′–3′)	Reverse Sequence (5′–3′)
Mouse *GFAP Cre*	ACTCCTTCATAAAGCCCTCG	ATCACTCGTTGCATCGACCG
Mouse *CD147 Loxp*	ATAGAAATGGGGGATGCTCTG	GGCTCTGTCTTCACTTGGGTT
Human *CXCL1*	ATGGCCCGCGCTGCTCTCTCC	GTTGGATTTGTCACTGTTCAG
Human *CD147*	ACTCCTCACCTGCTCCTTGA	GCCTCCATGTTCAGGTTCTC
Human *α1(I) collagen*	AACATGACCAAAAACCAAAAGT	CATTGTTTCCTGTGTCTTCTGG
Human *GAPDH*	GCACCGTCAAGGCTGAGAAC	ATGGTGGTGAAGACGCCAGT
Mouse *CD147*	TGGCAAGTATGTGGTGGTAT	GTGAGATGGTTTCCCGAGT
Mouse *α-SMA*	GTCCCAGACATCAGGGAGTAA	TCGGATACTTCAGCGTCAGGA
Mouse *desmin*	AACAGCCTCGGTTCCTTGAG	GACCTGAGGCTAAACAGGCG
Mouse *α1(I) collagen*	GCTCCTCTTAGGGGCCACT	CCACGTCTCACCATTGGGG
Mouse *CXCL1*	CACAGGGGCGCCTATCGCCAA	CAAGGCAAGCCTCGCGACCAT
Mouse *GAPDH*	AGGTCGGTGTGAACGGATTTG	TGTAGACCATGTAGTTGAGGTCA

## Abbreviations

α-SMA	α-smooth muscle actin
CXCL1	CXC chemokine-ligand-1
CCl_4_	Carbon tetrachloride
ECM	Extracellular matrix
ELISA	Enzyme-linked immunosorbent assay
FACS	Fluorescence activated cell sorting
GFAP	Glial fibrillary acidic protein
HSC	Hepatic stellate cell
MFI	Median fluorescence intensity
snc-RNA	Silencer-negative control siRNA
